# Reply to “Can femoral head necrosis induced by steroid therapy in patients infected with coronaviruses be reversed?”

**DOI:** 10.1038/s41413-020-00133-x

**Published:** 2021-01-07

**Authors:** Peixun Zhang, Jiabao Ju, Na Han, Baoguo Jiang

**Affiliations:** Department of Orthopedics and Trauma, Peking University People’s Hospital, Beijing, China

**Keywords:** Diseases, Calcium and phosphate metabolic disorders

The authors reply: We deeply appreciated your interest in our research. In our study, the conclusion that femoral head necrosis induced by large doses of steroid pulse therapy in SARS patients was partially reversible was based on the results of the volume of femoral head necrosis on MRI combined with the ARCO stage. The volume of osteonecrosis decreased significantly from 2003 (38.83% ± 21.01%) to 2005 (30.38% ± 20.23%) (*P* = 0.000 2), then declined slowly from 2005 to 2013 (28.99% ± 20.59%) and plateaued in 2018 (25.52% ± 15.51%). The ARCO stages of 17 limbs in ten patients stabilized or improved during follow-up, while the stages of six limbs in four patients reflected deterioration.

As Wang mentioned, case 11 and case 14 reversed their ARCO stages, while the association between ARCO stage and steroid use was unclear. In our study, the medical records of those patients were missing, and the doses and duration of steroid use were obtained with questionnaires; approximately half of the patients could not remember their cumulative dosage. The median steroid level was a rough estimation, which is one of the limitations of our study.

Since the start of the coronavirus disease 2019 (COVID-19) pandemic, there has been a debate on steroid use in critically ill patients. Guidance from the World Health Organization (WHO) on the management of patients with COVID-19 does not advise the use of corticosteroids.^1^ In a meta-analysis of corticosteroid use in patients with SARS, four studies provided data indicating that the use of steroids was harmful.^2^ Therefore, it is unlikely that patients with COVID-19 would benefit from large doses of corticosteroids. In our study, we reported the outcomes of pulmonary and femoral head lesions in healthcare workers with nosocomial SARS-CoV infections. Due to small sample size and missing data, our study provided limited evidence that can support prognostic predictions in patients with COVID-19.
